# WDR55 Is a Nucleolar Modulator of Ribosomal RNA Synthesis, Cell Cycle Progression, and Teleost Organ Development

**DOI:** 10.1371/journal.pgen.1000171

**Published:** 2008-08-29

**Authors:** Norimasa Iwanami, Tomokazu Higuchi, Yumi Sasano, Toshinobu Fujiwara, Vu Q. Hoa, Minoru Okada, Sadiqur R. Talukder, Sanae Kunimatsu, Jie Li, Fumi Saito, Chitralekha Bhattacharya, Angabin Matin, Takashi Sasaki, Nobuyoshi Shimizu, Hiroshi Mitani, Heinz Himmelbauer, Akihiro Momoi, Hisato Kondoh, Makoto Furutani-Seiki, Yousuke Takahama

**Affiliations:** 1 Division of Experimental Immunology, Institute for Genome Research, University of Tokushima, Tokushima, Japan; 2 Department of Chemical Science and Engineering, Graduate School of Engineering, Kobe University, Kobe, Japan; 3 Precursory Research for Embryonic Science and Technology, Japan Science and Technology Agency, Kawaguchi, Japan; 4 Department of Cancer Genetics, University of Texas, MD Anderson Cancer Center, Houston, Texas, United States of America; 5 Department of Molecular Biology, Keio University School of Medicine, Tokyo, Japan; 6 GSP Center, The Leading Institute of Keio University, Tsukuba, Japan; 7 Department of Integrated Biosciences, Graduate School of Frontier Sciences, University of Tokyo, Kashiwa, Japan; 8 Max Planck Institute for Molecular Genetics, Berlin, Germany; 9 Developmental Mutants Group, Kondoh Differentiation Signaling Project, Japan Science and Technology Agency, Kyoto, Japan; Stanford University School of Medicine, United States of America

## Abstract

The thymus is a vertebrate-specific organ where T lymphocytes are generated. Genetic programs that lead to thymus development are incompletely understood. We previously screened ethylnitrosourea-induced medaka mutants for recessive defects in thymus development. Here we report that one of those mutants is caused by a missense mutation in a gene encoding the previously uncharacterized protein WDR55 carrying the tryptophan-aspartate-repeat motif. We find that WDR55 is a novel nucleolar protein involved in the production of ribosomal RNA (rRNA). Defects in WDR55 cause aberrant accumulation of rRNA intermediates and cell cycle arrest. A mutation in WDR55 in zebrafish also leads to analogous defects in thymus development, whereas WDR55-null mice are lethal before implantation. These results indicate that WDR55 is a nuclear modulator of rRNA synthesis, cell cycle progression, and embryonic organogenesis including teleost thymus development.

## Author Summary

Medaka, *Oryzias latipes*, is a small freshwater fish that is useful for studies of forward and reverse genetics. The availability of various inbred strains is the distinct advantage of medaka over zebrafish, especially in studies of the immune system. The thymus is a primary immune organ that is unique to vertebrates and supports the generation of T lymphocytes. Defective thymus development tends to cause abnormal T lymphocyte development, resulting in immunodeficiency or autoimmunity. However, the molecular pathways underlying thymus development have not been fully uncovered. Here we report the positional cloning of a gene responsible for one of the thymus-defective medaka mutants. We find that the *hkc* phenotype is caused by a missense mutation in a gene encoding the previously uncharacterized protein WDR55. Our results indicate that WDR55 is a novel nucleolar modulator of rRNA biosynthesis, cell cycle progression, and vertebrate development of organs, including teleost thymus. These results not only provide evidence of the existence of a new mechanism of rRNA production but also demonstrate that the malfunction of WDR55 causes cell cycle arrest and developmental failure, including defective thymus development.

## Introduction

The thymus is a lymphopoietic organ that is unique to vertebrates and supports the generation of T lymphocytes. It is generated from the budding of third pharyngeal pouch endoderm and its interaction with ventrally migrating neural crest cells [1],[2]. Lymphoid precursor cells derived from hematopoietic stem cells immigrate to thymus primordium where they differentiate into mature T lymphocytes carrying diverse yet self-tolerant recognition repertoire [3],[4]. Defective thymus development tends to cause abnormal T lymphocyte development, resulting in immunodeficiency or autoimmunity [5]–[8]. Studies of patients and animal models have enabled identification of several genes required for thymus development. *Tbx1* is the gene responsible for DiGeorge syndrome, a condition characterized by cardiovascular, thymic, parathyroid, and craniofacial anomalies [9]–[11]. *Foxn1* is the gene responsible for severe immunodeficiency of *nude* phenotype in mouse and human, due to the lack of functional thymus and hair formation [12],[13]. Use of genetically modified mouse strains has enabled further identification of genes involved in thymus development [14],[1]. However, the molecular pathways underlying thymus development have not been fully uncovered.

We previously established a collection of ethylnitrosourea-induced medaka mutants that exhibited recessive defects in thymus organogenesis [15],[16]. Medaka, *Oryzias latipes*, is a small freshwater fish that is useful for studies of forward and reverse genetics [17]. Like zebrafish *Danio rerio*, medaka is one of the smallest vertebrate species equipped with an adaptive immune system that includes the thymus, T lymphocytes, and T-cell-mediated cellular immune responses, such as allograft rejection [18],[19]. The small size of the genome (800 Mb in medaka vs. 1700 Mb in zebrafish), along with the availability of various genomic resources, including a completed genome sequence, bacterial artificial chromosome library, and radiation hybrid maps, makes medaka a useful species for genomic analysis and genetic experiments, including transgenesis and morpholino antisense oligonucleotide-mediated gene knockdown [20]–[23]. The availability of various inbred strains is the distinct advantage of medaka over zebrafish [17], especially in studies of the immune system, such as the development and function of T lymphocytes. By screening ethylnitrosourea-induced mutants that covered approximately 60% of medaka genome, we established 22 mutant lines that have defects in immature-lymphocyte-specific *recombination activating gene 1 (rag1)* expression in the thymus. These medaka mutants would complement the panel of mutations affecting thymus organogenesis in zebrafish [24]–[26], since different spectrum of mutant phenotypes has been identified in medaka from that in zebrafish due to divergent functional overlap of related genes [16].

We report herein the positional cloning of a gene responsible for one of the thymus-defective medaka mutants, *hokecha (hkc)*, in which thymus primordium fails to accumulate lymphoid cells. We find that the *hkc* phenotype is caused by a missense mutation in a gene encoding previously uncharacterized protein WDR55 that carries the tryptophan-aspartate-repeat motif. We show that WDR55 modulates the nucleolar production of ribosomal RNA (rRNA) and *hkc* mutation causes a defect in the nucleolar localization of WDR55. The defect in WDR55 causes the accumulation of aberrant rRNA intermediates and cell cycle arrest. We also show that WDR55 mutation in zebrafish causes defective development of the thymus. Thus, the present results indicate that WDR55 is a novel nucleolar modulator of rRNA synthesis, cell cycle progression, and embryonic organogenesis, including teleost thymus development.

## Results

### *hkc* Is Defective in Development of Thymus Primordium

**Figure 1:**
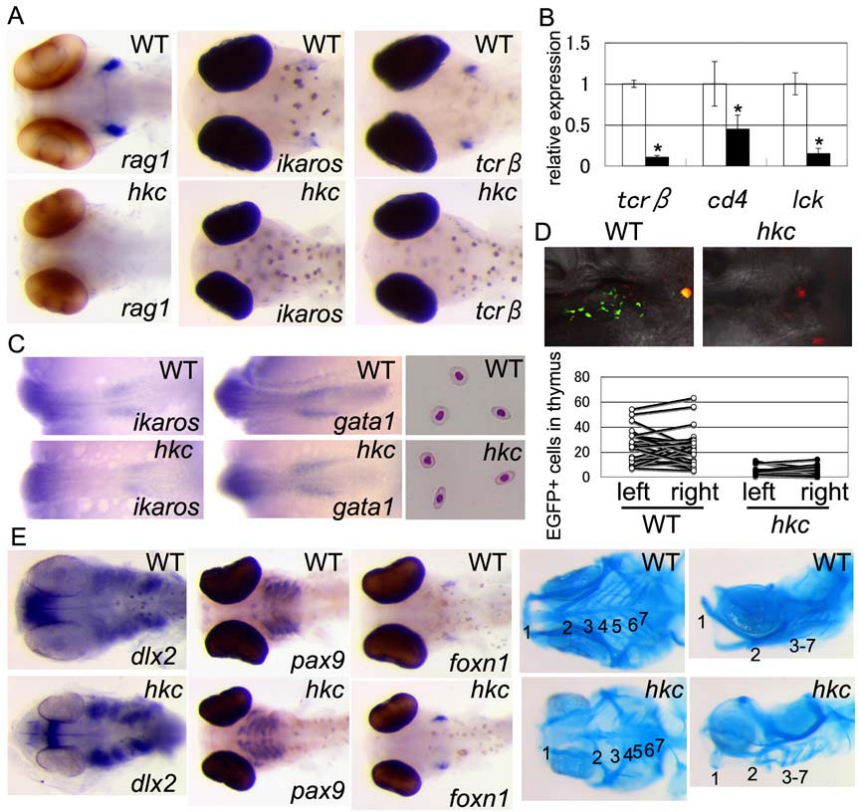
*hkc* is defective in thymus development. (A) Whole-mount *in situ* hybridization of 6-dpf wild-type (WT, top) and *hkc* (bottom) embryos using *rag1* (left), *ikaros* (middle), and *tcrβ* (right) probes. Ventral views are shown. Embryos were treated with H_2_O_2_ to bleach pigment cells in the *rag1* group. (B) Quantitative PCR analysis of indicated genes in whole bodies of 7-dpf WT (open bars) and *hkc* (closed bars) embryos. Expression in WT embryos was normalized to 1. Results represent averages and standard errors of four independent measurements. Asterisks, p<0.05. (C) Whole-mount *in situ* hybridization of WT (top) and *hkc* (bottom) embryos at stage 21 using *ikaros* (left) and *gata1* (center) probes. Dorsal views of posterior regions are shown. Right panels show Giemsa-May-Grunwald-stained red blood cells of 7 dpf WT (top) and *hkc* (bottom) embryos. (D) Transplantation of *rag1-EGFP* transgenic thymocytes into embryos. Top images show green fluorescence signals and red CMTMR signals in the thymus of WT (left) and *hkc* (right) recipients at 1 day after transplantation. Bottom plots indicate numbers of donor-derived EGFP^+^ cells in WT and *hkc* thymuses. Plots of left and right thymus in individual recipients are indicated. 15 and 11 recipients of WT and *hkc*, respectively, were analyzed. (E) Whole-mount *in situ* hybridization of WT (top) and *hkc* (bottom) embryos using *dlx2* (stage 26), *pax9* (6 dpf), and *foxn1* (5 dpf) probes. Ventral views are shown. Embryos were treated with H_2_O_2_ to bleach pigment cells in the *foxn1* group. Four panels on the right show Alcian blue staining of 9-dpf WT (top) and *hkc* (bottom) larvae. Ventral and lateral views are shown. Numbers indicate pharyngeal arches.

We previously established a medaka strain, *hokecha* (*hkc*), in which *rag1* expression in the thymus was undetectable [15]. T lymphocyte development in embryonic thymus of wild-type (WT) medaka could be visualized by whole-mount *in situ* hybridization of immature-lymphocyte-specific *rag1*, lymphocyte-specific *ikaros*, and T-lymphocyte-specific *T-cell receptor beta* (*tcrβ*), whereas none of these genes were detectable in the thymus of *hkc* mutants (Figure 1A). Unlike the thymus of wild-type medaka, accumulation of hematoxylin-rich lymphoid cells was not detectable at the pharyngeal region in *hkc* mutants (shown below). Systemic T lymphocytes were also undetectable in *hkc* by T-lymphocyte-specific genes *tcrβ*, *cd4*, and *lck* in whole embryos (Figure 1B).

T lymphocyte development in the thymus is initiated upon the migration of lymphoid precursor cells into thymus primordium [3]. To examine whether the development of lymphoid precursor cells is affected in *hkc*, we analyzed early hematopoiesis and pre-thymic lymphopoiesis in *hkc* embryos. Early hematopoiesis in the lateral mesoderm [27], which was detected by measuring erythrocyte-specific *gata1* and lymphocyte-specific *ikaros* expression, was unaltered in *hkc* mutant embryos at stage 21 (34 hours post fertilization (hpf)) (Figure 1C). In the course of the embryogenesis, hematopoiesis is relocated to intermediate cell mass and ventral wall of dorsal aorta, which is considered to correspond to the aorta-gonad-mesonephros region in mammals [28]-[30]. *ikaros* and *gata1* expression in the intermediate cell mass remained unaltered in *hkc* embryos at stage 23 (41 hpf) (data not shown). In addition, normally shaped red blood cells were generated in the circulation of *hkc* mutants (Figure 1C). These results indicate that hematopoiesis and early lymphopoiesis are detectable in *hkc*.

We next examined whether the development of thymus primordium might be affected in *hkc*. To this end, we transplanted EGFP-expressing immature lymphocytes into *hkc* embryos. EGFP-expressing immature lymphoid cells isolated from transgenic medaka expressing EGFP under the control of medaka *rag1* promoter [30] were injected into wild-type or *hkc* embryos via blood vessel and traced under a fluorescence microscope. We detected the migration of EGFP^+^ cells in the thymus of wild-type embryos and the remarkably reduced accumulation of EGFP^+^ cells in the thymus of *hkc* embryos (Figure 1D). These results indicate that the development of thymus primordium that attracts lymphoid precursor cells is defective in *hkc*. Nonetheless, it is possible that lymphoid precursor cells in *hkc* are also defective in colonization and/or development in the thymus.

Thymus primordium is generated through the interaction of third pharyngeal pouch endodermal cells with neural-crest-derived mesenchymal cells [1],[2]. The expression of *pax9* and *dlx2* that detect endodermal cells and neural-crest-derived cells, respectively, in pharyngeal pouch was slightly distorted but comparably detected in *hkc* embryos (Figure 1E). Moreover, the expression of thymic epithelial cell specific *foxn1* was detectable in *hkc* mutants (Figure 1E). On the other hand, pharyngeal arches in *hkc* were short and abnormally shaped (Figure 1E). We detected the thin seventh pharyngeal arch that we previously failed to detect [15] in *hkc* (Figure 1E).

These results indicate that *hkc* mutant medaka is unable to develop functional thymus primordium that is colonized by lymphoid precursor cells.

### Missense Mutation in *WDR55* Is Responsible for *hkc* Phenotype

**Figure 2:**
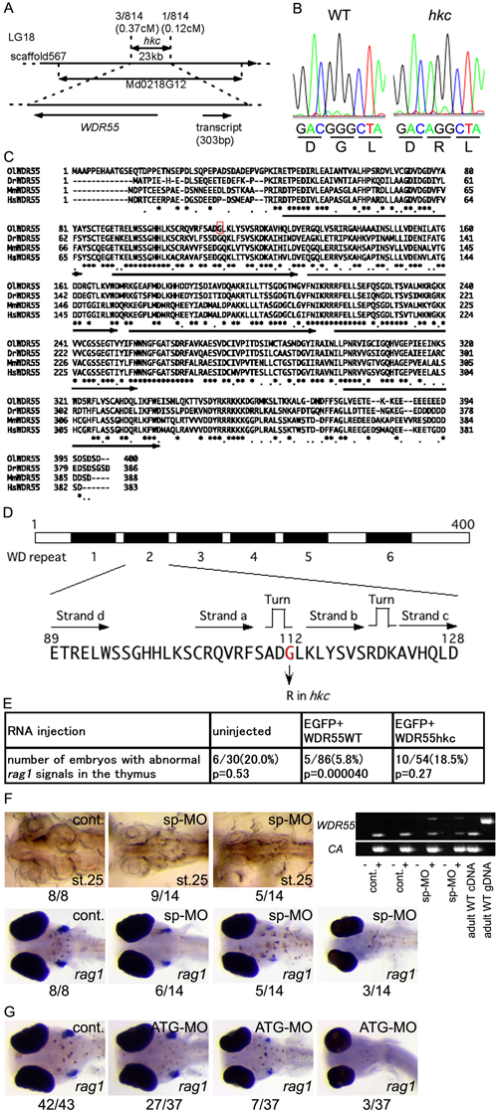
A missense mutation in *WDR55* is responsible for *hkc* phenotype. (A) *hkc* was mapped on linkage group (LG) 18 and confined in the 23 kb region on scaffold 567. Recombination rates of 407 *hkc* embryos from *hkc*/+ (cab-derived)×kaga crosses and cM distances from *hkc* to neighboring markers are shown. These markers are located in a single BAC Md0218G12. Indicated two genes were predicted in the mapped region. (B) Sequences within the *WDR55* coding region of WT (left) and *hkc* (right) genomic DNA and predicted amino acid sequences. (C) Predicted amino acid sequence of medaka (Ol) *WDR55* was compared with zebrafish (Dr), mouse (Mm), and human (Hs) *WDR55* sequences. Asterisks and dots indicate residues that are shared among all four species and three species, respectively. Arrows indicate WD repeat domains. Red box indicates glycine residue that is replaced with arginine in *hkc* mutants. (D) Predicted structure of medaka WDR55 protein. Filled boxes indicate WD repeat domains. Amino acid residues and predicted secondary structures of the second WD repeat domain are also shown. (E) 1-cell-stage embryos from *+*/*hkc*×*+*/*hkc* matings were injected with WT- or *hkc*-derived *WDR55* mRNA and *EGFP* mRNA, and whole-mount *in situ* hybridization using *rag1* probe was carried out at 6 dpf. P-values were calculated using κ-square test. (F) 50 µM of morpholino that blocks splicing of *WDR55* (sp-MO) was injected into WT embryos. Three images appearing on top left show dorsal views of control embryos (cont.) and morphants (sp-MO) at stage 25. Four images appearing at the bottom show ventral views of control (cont.) and 5-dpf morphants (sp-MO) hybridized with *rag1* probe. Numbers below images indicate the numbers of embryos showing phenotypes of the images over the numbers of total embryos examined. Top right images show ethidium bromide (EtBr)-stained gels of RT-PCR products for two *WDR55* exons neighboring the position of splicing-inhibiting morpholino. Total RNAs from two individual control embryos (cont.) and two individual morphants (sp-MO) at stage 25 were examined. cDNA was synthesized in the absence (−) or presence (+) of reverse transcriptase. Adult WT cDNA and genomic DNA were also amplified. cDNA for *cytoplasmic actin* (CA) was amplified to verify the quality of cDNA synthesized. (G) 50 µM of morpholino that was designed to block translation of *WDR55* mRNA was injected into WT embryos. Three images show ventral views of control (cont.) and 6-dpf morphants (ATG-MO) hybridized with *rag1* probe. Numbers below images indicate the numbers of embryos showing phenotypes of the images over the numbers of total embryos examined.

Positional cloning was carried out to identify the mutation responsible for the *hkc* phenotype. Linkage analysis mapped *hkc* gene within the 23 kb region on scaffold 567 (covered by a single BAC clone Md0218G12) of linkage group (LG) 18 (Figure 2A). According to gene prediction by Genscan, this region contained two genes, *WDR55* (EST clone MF01SSB013N12) and an unnamed transcript that contained presumptive 303 bp coding region (Figure 2A). We found that *hkc* allele carried a guanine (G) to adenine (A) point mutation in the coding region of *WDR55*, whereas no mutations were found in the 303 bp transcript (Figure 2B). The predicted open reading frame of *WDR55* encodes a 400 amino acid protein containing six tryptophan-aspartate-repeat (WDR) motifs. The deduced amino acid sequence was 58%, 59%, and 66% identical to human, mouse, and zebrafish WDR55, respectively (Figure 2C). BLAST search found no other *WDR55*-like loci in the genome of medaka, zebrafish, mouse, and human (data not shown).

The WDR motif is shared among various proteins involved in signal transduction, cell cycle control, and transcriptional regulation [31]. The point mutation in *hkc* caused a glycine to arginine substitution at the 112th amino acid residue that was projected from β sheets in the second WDR motif, according to structural prediction by Smith et al. (1999) [32] (Figures 2B, 2C, and 2D). Injection of wild-type *WDR55* mRNA into homozygous *hkc* embryos rescued *hkc* phenotypes including defective *rag1* expression in the thymus, whereas injection of the same amount of *hkc WDR55* mRNA failed to rescue *hkc* phenotypes (Figure 2E). In contrast, more than half of wild-type medaka embryos that were administered morpholino antisense oligonucleotide to block the splicing of *WDR55* and reduce spliced *WDR55* mRNA (Figure 2F) phenocopied defective thymus development and small eye size found in *hkc* mutants (Figure 2F). Another WDR55-specific morpholino that was designed to hybridize a start-codon-containing sequence and to block translation of *WDR55* mRNA also caused defective thymus development in wild-type medaka (Figure 2G). These results indicate that the G to A point mutation in *WDR55* gene is responsible for the thymus-defective phenotype of *hkc* mutants.

### WDR55 Modulates Nucleolar rRNA Production and Cell Cycle Progression

**Figure 3:**
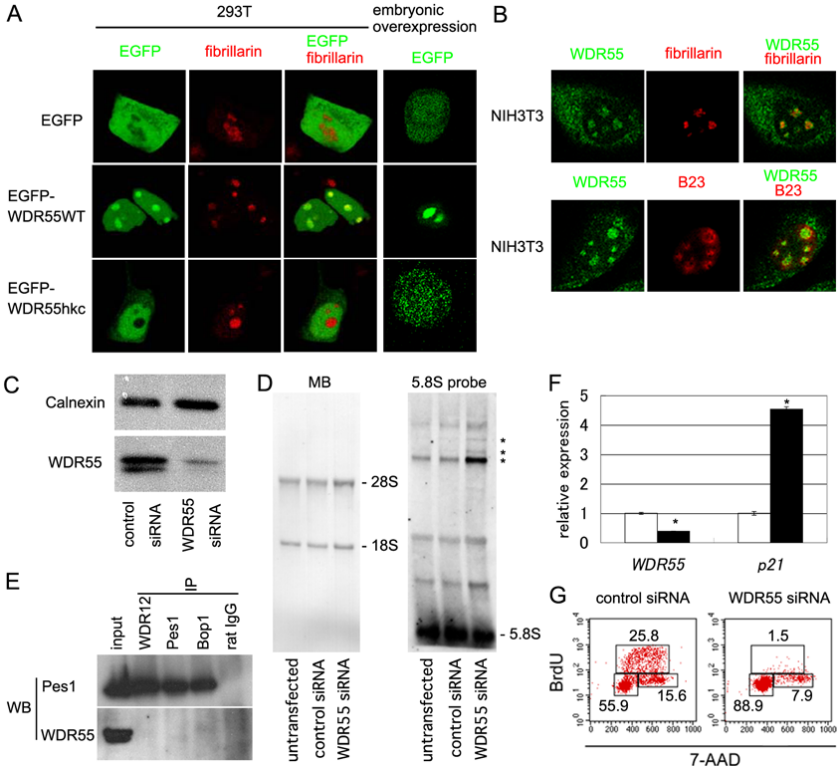
WDR55 modulates nucleolar rRNA production and cell cycle progression. (A) Intracellular localization of transfected EGFP, EGFP-WDR55WT, and EGFP-WDR55hkc (green) along with co-transfected t-HcRed1-fibrillarin in 293T cells. Single fluorescence images and merged images are shown. Rightmost panels show images of single cells isolated from stage-19 embryos that were administered at 1-cell stage with mRNAs of indicated genes. (B) Localization of endogenous WDR55 in NIH3T3 cells identified by antibody staining. Single fluorescence images and merged images of WDR55 (green) and fibrillarin (red, top panels) or B23 (red, bottom panels) are shown. (C) Western blotting of whole cell lysates of control or WDR55-siRNA-transfected NIH3T3 cells at 44 hours after transfection using anti-calnexin or anti-WDR55 antibody. (D) Detection of rRNA processing intermediates by Northern blot hybridization with 5.8S rRNA probe of total RNA (0.5 µg per lane) isolated from untransfected, control-siRNA-transfected, and WDR55-siRNA-transfected NIH3T3 cells. Asterisks indicate accumulation of processing intermediates in NIH3T3 cells transfected with WDR55-siRNA. An image of methylene blue (MB) staining is also shown (left). The amount of housekeeping *glyceraldehyde-3-phosphate dehydrogenase* mRNA measured by quantitative RT-PCR was not significantly different among same-weight total cellular RNAs isolated from untransfected, control-siRNA-transfected, and WDR55-siRNA-transfected cells. Along with the data showing that the intensities of the bands for 28S, 18S, and 5.8S rRNAs were comparable among these three groups of cells, these results suggest that the total amounts of rRNAs were comparable among these three groups of cells. (E) Immunoprecipitation (IP) of U2OS cell lysates using anti-WDR12, anti-Pes1, and anti-Bop1 antibodies, or normal rat IgG. 2.5% of input lysate before IP was also electrophoresed. Western blot (WB) detection is shown for Pes1 (top) and WDR55 (bottom). (F) Quantitative RT-PCR analysis of indicated genes in control (open bars) and WDR55-siRNA-transfected (closed bars) NIH3T3 cells. Expression levels in control cells were normalized to 1. Results represent averages and standard errors of three independent measurements. Asterisks, p<0.05. (G) Cell cycle analysis of control (left) and WDR55-siRNA-transfected NIH3T3 cells by BrdU and 7-AAD staining. Numbers indicate frequency of cells in indicated squares. Lower left box, upper box, and lower right box show cells in G1 phase, S phase, and G2/M phase, respectively. Representative results of three independent experiments are shown.

Because the function of WDR55 was previously unknown, we first examined the intracellular localization of medaka WDR55 tagged with EGFP and expressed in human 293T cells. We found that EGFP fused with wild-type WDR55 was chiefly condensed in the nucleolus, as determined by the merged localization with co-transfected t-HcRed1-fibrillarin (Figure 3A). In contrast, EGFP fused with *hkc*-mutant WDR55 was excluded from the nucleolus (Figure 3A). Similar results showing the nucleolar accumulation of EGFP fused with wild-type WDR55 and the exclusion of EGFP fused with *hkc*-mutant WDR55 from the nucleolus were obtained upon transfection into mouse NIH3T3 cells (data not shown). Medaka somatic cells expressing EGFP fused with WDR55, but not *hkc*-mutant WDR55, showed intra-nuclear dot-like localization of EGFP (Figure 3A). Antibody detection of mouse WDR55 in NIH3T3 cells further showed that endogenous WDR55 was detectable in the nucleolus as well as the cytoplasm (Figure 3B). Endogenous WDR55 detected in the nucleolus co-localized with fibrillarin, which was enriched in the dense fibrillar component of the nucleolus [33], rather than with B23 nucleophosmin, which was enriched in the peripheral granular component of the nucleolus (Figure 3B). These results indicate that WDR55 is a nucleolar protein enriched in the dense fibrillar component and *hkc* mutation of WDR55 perturbs its nucleolar localization.

The dense fibrillar component in the nucleolus is the place where early processes of ribosome biosynthesis take place [34]. In order to examine the possible involvement of WDR55 in ribosome biosynthesis, NIH3T3 cells were transfected with siRNA specific for mouse WDR55 and examined for rRNA processing. siRNA transfection specifically reduced WDR55 expression in NIH3T3 cells (Figure 3C). We found that incompletely processed rRNA precursors as detected by hybridization with a 5.8S rRNA probe more strongly accumulated in WDR55-siRNA-transfected NIH3T3 cells than in control-siRNA-transfected or untransfected NIH3T3 cells (asterisks in Figure 3D). However, mature 5.8S, 18S, and 28S rRNAs were produced in WDR55-siRNA-transfected cells (Figure 3D). There were no significant differences in overall ribosome profiles between control and WDR55-siRNA-transfected NIH3T3 cells (data not shown). These results indicate that incompletely processed rRNA intermediates are accumulated by defective WDR55 expression.

It was previously shown that similar to WDR55, such WDR-motif-carrying proteins as WDR12 and Bop1 are localized in the nucleolus where they regulate rRNA processing [35]. However, unlike WDR12 and Pes1, WDR55 was not co-immunoprecipitated with WDR12, Bop1, or Pes1 (Figure 3E), suggesting that WDR55 is a novel modulator of rRNA production without physical association with the PeBoW complex that contains Pes1, Bop1, and WDR12.

At 2 days after WDR55-siRNA transfection into NIH3T3 cells, the expression of *p21^Waf1/Cip1^*, a gene that is regulated by p53 and controls cell cycle [36],[37], was significantly increased, while that of *WDR55* mRNA was significantly decreased compared to those in control-siRNA-transfected cells (Figure 3F). Accordingly, the frequency of cells in S phase was markedly decreased in WDR55-siRNA-transfected cells (Figure 3G), indicating that the defective expression of WDR55 results in cell cycle arrest at G1 phase. Two other WDR55-siRNAs that reduced *WDR55* mRNA expression less strongly than that used in Figures 3C, D, F, G increased p21 expression and decreased the number of S-phase cells less strongly (data not shown). These results demonstrate that WDR55 modulates nucleolar rRNA production and the defective expression of WDR55 affects p53 activation and cell cycle progression.

### Accumulation of Incompletely Processed rRNA in *hkc* Mutant

**Figure 4:**
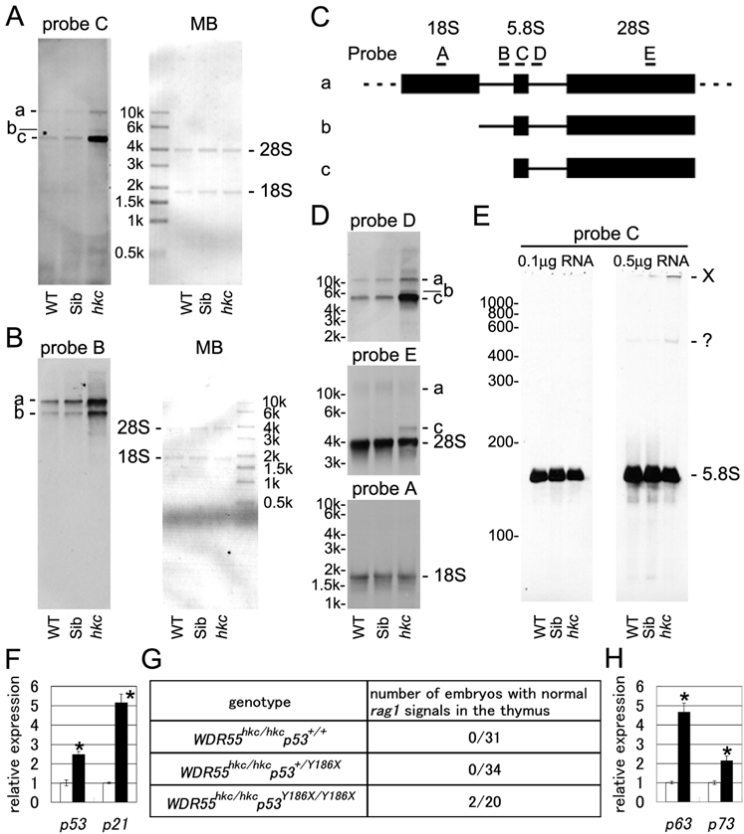
Defective rRNA processing and p53 activation in *hkc* mutants. (A–E) Detection of rRNA processing intermediates by Northern blot hybridization. Total cellular RNAs (0.5 µg per lane for probes A, C, and E; 0.1 µg per lane for probes B and D) in 7-dpf embryos from WT, siblings (Sib), and *hkc* mutants were electrophoresed in agarose gels and was hybridized with indicated probes (panels A, B, and D). Images of methylene blue (MB) staining are also shown. Indicated amount of total cellular RNAs in 7-dpf embryos from WT, siblings (Sib), and *hkc* mutants were electrophoresed in polyacrylamide gel and hybridized with probe C (panel E). Predicted structures of intermediates a-c and positions of probes A–E are drawn (panel C). In panel E, the *hkc* lane shows an increase in slowly electrophoresed signal (designated as X), perhaps corresponding to the mixture of the intermediates described in panel C. The amount of housekeeping *cytoplasmic actin* mRNA measured by quantitative RT-PCR was not significantly different among same-weight total cellular RNAs isolated from WT, Sib, and *hkc* mutants. Along with data showing that the intensities of the bands for 28S, 18S, and 5.8S rRNAs were comparable among these three groups of cells, these results suggest that the total amounts of rRNAs were comparable among these three groups of the cells. (F) Quantitative RT-PCR analysis of indicated genes in whole bodies of 7-dpf WT (open bars) and *hkc* mutants (closed bars). Results represent averages and standard errors of four independent measurements. Asterisks, p<0.05. (G) Phenotypes of *WDR55^hkc/hkc^p53^Y186X/Y186X^* embryos. Whole-mount *in situ* hybridization of *rag1* was carried out with 5- to 7-dpf embryos obtained from *WDR55^+/hkc^p53^Y186X/Y186X^*×* WDR55^+/hkc^p53^Y186X/Y186X^* crosses or *WDR55^+/hkc^p53^+/Y186X^*× *WDR55^+/hkc^p53^+/Y186X^* crosses. (H) Quantitative RT-PCR analysis of indicated genes in whole bodies of 7-dpf WT (open bars) and *p53^Y186X/Y186X^* mutants (closed bars). Results represent averages and standard errors of four independent measurements. Asterisks, p<0.05.

Northern blot hybridization of total RNA from 7 days post-fertilization (dpf) whole medaka embryos showed that incompletely processed rRNA intermediates accumulated in *hkc* mutants but not their siblings or wild-type medaka embryos (Figure 4A–F). Hybridization using probe C that was designed within the 5.8S rRNA sequence showed that rRNA processing intermediates ‘a’, ‘b’, and ‘c’ were more strongly detectable in *hkc* mutants than in siblings or wild-type medaka embryos (Figure 4A). Intermediates ‘a’ and ‘b’ but not ‘c’ accumulated in *hkc* mutants were detectable by probe B that was designed within the internal transcribed spacer 1 (ITS1) sequence between 18S and 5.8S rRNA sequences (Figure 4B), suggesting that intermediates ‘a’ and ‘b’ presumably corresponded to ITS1-containing intermediates with and without 18S rRNA, respectively, and intermediate ‘c’ presumably contained 5.8S rRNA, ITS2, and 28S rRNA sequences without ITS1 sequence (Figure 4C). Indeed, probe D that was designed within the ITS2 sequence between 5.8S and 28S rRNA sequences visualized the accumulation of intermediates ‘a’, ‘b’, and ‘c’ (Figure 4D). Nonetheless, *hkc* mutants produced mature 5.8S, 18S, and 28S rRNAs (probes C, A, and E, respectively; Figures 4D and E). These results indicate that similar to WDR55-siRNA-transfected NIH3T3 cells, *hkc* mutation of WDR55 affects rRNA processing and induces the accumulation of incompletely processed rRNA intermediates *in vivo*.

The expression of *p53* and *p21^Waf1/Cip1^* in whole embryos at 7 dpf was significantly higher in *hkc* mutants than in wild-type medaka (Figure 4F), suggesting that WDR55 mutation in *hkc* causes p53 activation and results in developmental defects. However, we did not detect restoration of *rag1*-expressing cells in the thymus or normal-sized eyes at 7 dpf in *hkc* mutants that also lacked functional p53 by Y186X truncation [38] (Figure 4G). It was previously suggested that the expression of p53 family molecules, such as p63 and p73, could be elevated in the absence of p53 and the elevated p63 and/or p73 might compensate for the loss of p53 [39],[40]. Indeed, we found that *p53^Y186X/Y186X^* mutant medaka embryos showed significantly elevated expression of *p63* and *p73* (Figure 4H). Thus, it was possible that defective thymus development in *WDR55^hkc/hkc^ p53^Y186X/Y186X^* double mutants might be signaled via p63 and/or p73. These results suggest that *hkc* mutation causes the accumulation of incompletely processed rRNA intermediates and activates p53 family molecules.

### How Does *hkc* Mutation Result in Defects in Thymus Development?

**Figure 5:**
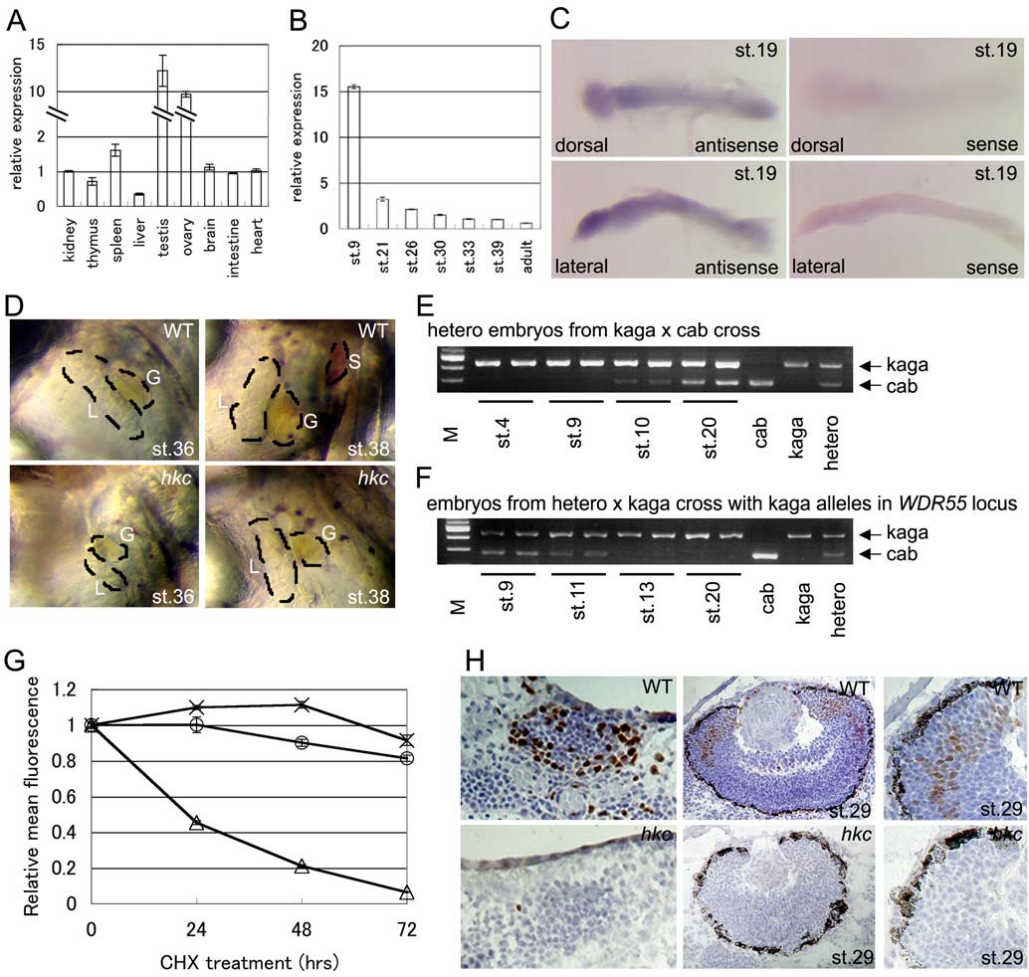
How does *hkc* mutation result in defects in thymus development? (A) Quantitative RT-PCR analysis of *WDR55* in indicated organs of adult WT medaka. The expression in kidney was normalized to 1. Shown are averages and standard errors of three independent measurements. (B) Quantitative RT-PCR analysis of *WDR55* in whole bodies of WT medaka at indicated stages. The expression at stage 39 was normalized to 1. Shown are averages and standard errors of four independent measurements. (C) Whole-mount *in situ* hybridization of WT embryos at stage 19 using *WDR55* antisense (left) and sense (right) probes. Dorsal views (top) and lateral views (bottom) are shown. (D) Left ventrolateral views of WT (top) and *hkc* (bottom) embryos at stage 36 (left) and stage 38 (right). L, liver; G, gall bladder; S, spleen. (E) cDNAs from whole medaka embryos at indicated stages from the mating of kaga females with cab males were amplified for *WDR55*, followed by cab genotype-specific restriction digestion with BstNI. cDNAs from two individual embryos were analyzed for each stage. cDNAs from adult cab, kaga, and their heterozygotes are shown on the rightmost three lanes. M: size marker. (F) Cab/kaga heterozygous females and kaga males were mated and embryos were selected for individuals carrying kaga-derived *WDR55* loci at both alleles. cDNAs from whole medaka embryos at indicated stages were amplified for *WDR55*, followed by cab genome-specific restriction digestion. (G) Stability of WDR55 proteins. 293T cells were transfected with EGFP (×), EGFP-WDR55WT (○), or pZsProSensor-1 (△) and treated with cycloheximide (CHX) for indicated periods. Fluorescence intensity was measured with a flow cytometer and relative mean fluorescence intensity was plotted. Shown are averages and standard errors of three independent measurements. (H) Detection of BrdU incorporation in WT (top) and *hkc* (bottom) embryos. Brown, BrdU; blue, hematoxylin. Thymuses of 7-dpf embryos (left) and eyes at stage 29 (middle panels and high-magnification images in right panels) are shown.

We then addressed how *hkc* mutation resulted in defects in thymus development rather than systemic failure of the development at much earlier stages. To do so, we initially examined *WDR55* expression in adult and embryonic medaka. We found that the expression of *WDR55* was detectable in every organ, including the thymus, of adult medaka by quantitative RT-PCR analysis (Figure 5A). Among adult medaka tissues, *WDR55* expression was most prominent in reproductive organs, such as testis and ovary (Figure 5A). During embryogenesis, *WDR55* expression was stronger in early embryonic stages than in late ones (Figure 5B) and widespread in embryonic body, as detected by whole-mount *in situ* hybridization (Figure 5C). These results indicate that *WDR55* is expressed ubiquitously in medaka and is not specific to the thymus, suggesting that the defects in *hkc* might not be limited to the thymus.

In fact, we found that in addition to the thymus, the spleen was absent in 6-dpf and 7-dpf *hkc* mutants (Figure 5D). Similar to the head and the eyes, the liver and the gall bladder were smaller in *hkc* mutants than in wild-type medaka (Figure 5D). Pharyngeal arches were abnormally shaped and lower jaws were malformed in *hkc* mutants (Figure 1E). On the other hand, hematopoiesis and development of the gills and the gut, as well as body axis formation, were not disturbed at 7 dpf. Nevertheless, *hkc* mutants were lethal between 8 and 10 dpf. These results indicate that *hkc* mutation of WDR55 causes systemic and lethal failure of medaka development by 10 dpf, and several organs including the thymus are more severely affected than other organs during embryogenesis of *hkc* mutants before lethality.

Then, we examined how the development in *hkc* mutants was not arrested at much earlier stages but could be sustained until 10 dpf. It is known that zygotic transcription in medaka begins around mid-blastula transition at 8.25 hours post-fertilization (hpf) or stage 11 [41], and early development at least before this transition is regulated by maternally inherited mRNA and proteins [42]. Indeed, embryonic transcription of *WDR55*, as revealed by measurement of paternal allele-specific mRNA in heterozygous embryos, was first detectable in early blastula at 6.5 hpf or stage 10 (Figure 5E), whereas *WDR55* mRNA specific for maternally inherited allele was detectable until late blastula at stage 11 (Figure 5F). On the other hand, measurement of the decay of WDR55-EGFP fusion protein in cycloheximide-treated 293T cells indicated that the half-life of WDR55-EGFP fusion protein was substantially longer than 3 days and estimated to be 7 to 10 days in the cells (Figure 5G). These results suggest that maternally inherited WDR55 mRNA and proteins are present in medaka embryos and normal WDR55 proteins derived from female parents may support the embryogenesis of *hkc* mutants for up to 10 days.

We further wanted to address how *hkc* embryos exhibited the defects in several organs such as the thymus more severely than in other organs. To do so, we examined the status of cell proliferation in medaka embryonic tissues. Many large proliferating cells detected by bromodeoxyuridine (BrdU) labeling were found in the thymus of wild-type medaka at 7 dpf (Figure 5H). Many cells in the eyes were also proliferating in 3 dpf medaka embryos (Figure 5H). However, these proliferating cells in the thymus or the eyes were barely detectable in stage-matched *hkc* mutants (Figure 5H). In addition to the thymus and the eyes, we detected active proliferation in the intestine and the gills, as visualized by BrdU incorporation (data not shown). Together, these results suggest that cells generating several organs including the thymus and the eyes undergo massive cell cycle progression, so that the cells that form these organs of *hkc* mutant embryos would rapidly consume maternally inherited normal WDR55 proteins, thereby exhibiting severe defects in organogenesis. Nonetheless, it is unclear whether all of the tissues affected in *hkc* mutants exhibit rapid proliferation.

### Zebrafish WDR55 Mutant Shows Defect in Thymus Development

**Figure 6:**
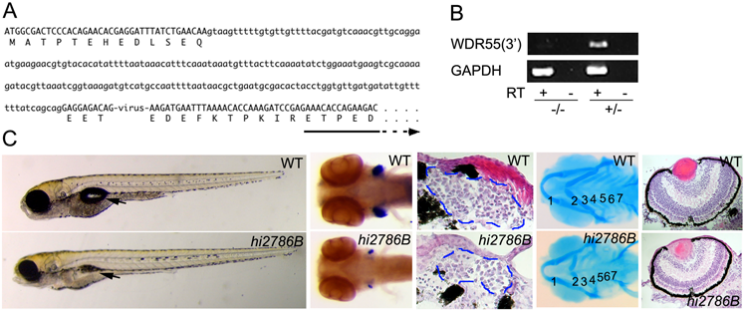
Zebrafish WDR55 mutant shows defect in thymus development. (A) Retroviral insertion in WDR55 locus of zebrafish *hi2786B* mutants. Uppercase letters indicate exons with predicted amino acids. Arrow indicates WDR domain. (B) Transcription of *WDR55* 3′ region and GAPDH in *hi2786B* (−/−) and heterozygous (+/−) zebrafish larvae at 6 dpf. cDNA was synthesized in the absence (−) or presence (+) of reverse transcriptase (RT). (C) Phenotypes of WT (top) and *hi2786B* (bottom) zebrafish. Far left panels show lateral views of 6-dpf larvae. Arrows indicate swim bladder. Second panels from the left show whole-mount *in situ* hybridization of 6-dpf larvae using *rag1* probe. Ventral views are shown. Embryos were treated with H_2_O_2_ to bleach pigment cells. Middle panels show hematoxylin-eosin (HE) staining of transverse sections at 6 dpf. Broken blue lines indicate the thymus. Second panels from the right show Alcian blue staining of 6-dpf larvae. Ventral views are shown. Numbers indicate pharyngeal arches. Far right panels show HE staining of transverse sections of the eyes at 6 dpf.

Zebrafish strain *hi2786B* was previously established as one of the lethal mutants caused by random retroviral insertion, and the retrovirus in *hi2786B* allele was found to be inserted within zebrafish *WDR55* locus [43]. We found that the retrovirus in *hi2786B* allele was inserted in the *WDR55* coding region before the first WDR motif (Figure 6A) and the 3′ region of *WDR55* open reading frame was not transcribed in *hi2786B* embryos (Figure 6B), suggesting that *hi2786B* is a WDR55-null mutant. Until lethality around 10 to 12 dpf, overall body formation including head and tail appeared intact in *hi2786B* mutants (Figure 6C). However, similar to *hkc* mutants in medaka, thymus size was remarkably reduced and the pharyngeal arches were malformed in *hi2786B* mutants (Figure 6C). Also, similar to *hkc* mutants, the eyes of *hi2786B* mutants were small (Figure 6C). Swim bladder in *hi2786B* mutants was small as well (Figure 6C). These results indicate that WDR55 deficiency in *hkc* medaka and *hi2786B* zebrafish results in similar defects in development, including defective formation of the thymus.

### WDR55 Deficiency in Mouse Causes Developmental Arrest before Implantation

**Figure 7:**
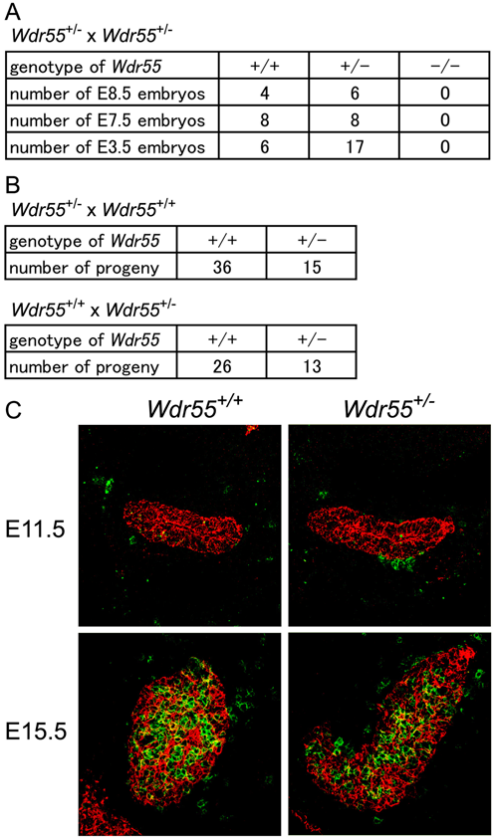
WDR55 deficiency in mouse causes developmental arrest before implantation. (A) Genotype of mouse embryos from *Wdr55^+/−^*× *Wdr55^+/−^* crosses at indicated embryonic days. (B) Genotype of mouse progenies from the mating of *Wdr55^+/−^* females×*Wdr55^+/+^* males (top) and *Wdr55^+/+^* females×*Wdr55^+/−^* males (bottom). (C) Thymus development in *Wdr55^+/−^* embryos. Cryosections of developing thymus in *Wdr55^+/+^* (left) and *Wdr55^+/−^* (right) embryos at E11.5 (top) and E15.5 (bottom) were stained with anti-CD45 (green) and anti-cytokeratin (red) antibodies.

WDR55 in mice was also expressed systemically, and WDR55-deficient mice previously established by targeted mutation were found to die before E9.5 [44]. We examined earlier development of WDR55-deficient mice and found that WDR55-deficient embryos disappeared as early as E3.5 (Figure 7A), indicating that WDR55 deficiency causes early arrest of mouse development before implantation. WDR55^+/−^ progenies were derived from either mating WDR55^+/−^ females with wild-type males or mating wild-type females with WDR55^+/−^ males, although the number of heterozygote progenies obtained from these crosses was reduced (Figure 7B). Thus, spermatogenesis and oogenesis were not severely arrested without WDR55. Haploinsufficiency in WDR55^+/−^ mice did not cause defects in development including thymus formation during embryogenesis (Figure 7C). These results indicate that WDR55 deficiency in mouse causes developmental arrest before implantation.

## Discussion

The present study revealed that a medaka *hkc* mutation that affects the development of functional thymus primordium and causes lethality by 10 dpf is due to a missense point mutation in the gene encoding WDR55, a novel protein. We found that WDR55 is a nucleolar protein ubiquitously expressed in various organs, and *hkc* mutation of WDR55 perturbs its nucleolar localization and causes the accumulation of incompletely processed rRNA intermediates. We also found that the defective expression of WDR55 affects cell cycle progression and *hkc* mutation activates the p53 pathway. A zebrafish WDR55 mutant showed similar developmental defects, including the lack of thymus formation and lethality by 12 dpf, whereas WDR55 deficiency in mouse caused much earlier developmental arrest before implantation. These results indicate that WDR55 modulates rRNA production, cell cycle progression, and vertebrate development, including teleost thymus organogenesis.

### Role of WDR55 in rRNA Production in Nucleolus

Recombinant gene mapping and DNA sequencing as well as mRNA-mediated rescue of *hkc* embryos and morpholino antisense oligonucleotide mediated phenocopy in wild-type embryos identified a point mutation in *WDR55* that is responsible for defective thymus formation in *hkc* mutants. WDR55 is a member of a large family of proteins that contain 4–16 tryptophan-aspartate-repeat (WDR) motifs, which consist of 40-60 amino acids with glycine-histidine dipeptide at the 11th to 24th residues from the N terminus and tryptophan-aspartate dipeptide at the C terminus [31]. Crystal structure analysis of G-protein β subunit, a WDR-motif-containing protein, has indicated that a WDR motif forms one blade of β propeller [31],[32],[45]. The WDR motif is implicated in protein-protein interaction, and WDR-motif-containing proteins have a variety of functions, including signal transduction, cell cycle regulation, and RNA synthesis [31].

The present study describes the function of a previously uncharacterized WDR-motif-containing protein, WDR55. Fluorescence localization of EGFP fusion protein containing medaka WDR55 sequence as well as antibody detection of endogenously expressed mouse WDR55 in the cells indicates that WDR55 proteins are localized in the nucleolus and the cytoplasm. Interestingly, *hkc* mutation that causes arginine substitution of glycine, which is presumably localized in the protruded loop between the second and third β sheets in the second propeller [31], causes the exclusion of WDR55 from the nucleolus. It is unclear whether this mutation affects nucleolar localization of WDR55 by specifically altering the capability of WDR55 for nucleolar localization or disrupting the overall structure of the protein. Nevertheless, aberrant localization of WDR55 by *hkc* mutation suggests that nucleolar localization is pivotal for WDR55 to exert its function. Within the nucleolus, WDR55 is enriched in the dense fibrillar component where early processes of rRNA biosynthesis take place [34].

Our results show that both *hkc* mutation of medaka WDR55 *in vivo* and siRNA-mediated reduction of mouse WDR55 expression in cell culture affect rRNA processing in the nucleolus. WDR55 reduction does not severely impair the production of 18S, 5.8S, and 28S rRNAs; rather it induces excessive accumulation of rRNA processing intermediates. Previous studies have shown that many proteins are involved in rRNA processing [34]. Among them, several proteins containing WDR motifs are known to be involved in rRNA synthesis in the nucleolus. For example, Rsa4p is involved in rRNA processing and transport of large ribosomal subunits [46], and WDR12 associated with another WDR-motif-containing protein, Bop1, along with Pes1 is involved in the processing of 5.8S/28S rRNA [35]. It is interesting to note the similarity of WDR55 to this PeBoW complex consisting of Pes1, Bop1, and WDR12 in terms of structures sharing WDR motifs and functions in 5.8S/28S rRNA processing. However, the results of co-immunoprecipitation experiments do not support the possibility that WDR55 is an additional member of the PeBoW complex. Thus, WDR55 is a novel modulator of rRNA synthesis in the nucleolus. It is unclear whether WDR55 is involved directly in rRNA processing, indirectly in the clearance of rRNA processing intermediates, or in rRNA processing/synthesis via other mechanisms. Nonetheless, it is possible that WDR55 may participate in linking rRNA production to cell cycle regulation.

We found that siRNA-mediated reduction of WDR55 expression in a mouse cell line causes p53 activation and cell cycle arrest, and that *hkc* mutation of medaka WDR55 *in vivo* analogously causes p53 activation and developmental defects. It is well known that nucleolar accumulation of rRNA intermediates activates the p53 pathway [47]–[49]. Accordingly, dominant-negative WDR12 accumulates rRNA processing intermediates and arrests cell cycle through p53 activation [34]. Also, a zebrafish *pes1* mutant exhibits small eyes and developmental failure in multiple organs [50]. Perhaps through a similar mechanism of nucleolar stress, either siRNA-mediated reduction or *hkc* mutation of WDR55 accumulates rRNA intermediates that may activate p53 family molecules and thereby lead to cell cycle arrest.

It is unclear whether developmental arrest in *hkc* mutants is indeed mediated by p53 activation, since our results show that p53 deficiency does not rescue developmental defects in *hkc* mutants. Since we detected significant increases in the expression of p63 and p73 in p53-deficient mutant medaka, it is possible that the elevated p63 and/or p73 may compensate the loss of p53 in p53-deficient medaka. Thus, it is possible that nucleolar stress in *hkc* mutants activates p53 family molecules that relay signals for cell cycle arrest and developmental defects.

### Role of WDR55 in Teleost and Mouse Development

WDR55 is ubiquitously expressed in various organs in medaka and mouse. We showed that *hkc* mutation of WDR55 causes lethal failure of medaka development by 10 dpf and several organs including the thymus are severely affected during embryogenesis of *hkc* mutants before lethality. Our results support the possibility that maternally inherited WDR55 mRNA and proteins may support the embryogenesis of *hkc* mutants. Since cells generating several organs including the thymus are shown to undergo massive cell cycle progression, it is possible that the cells that form these organs of *hkc* mutant embryos may rapidly consume maternally inherited normal WDR55 proteins, thereby exhibiting arrest in cell cycle progression and severe defects including failure in thymus organogenesis.

However, it is also possible that WDR55 may be somehow associated with molecules that are specifically expressed in several organs including the thymus, and the defect in WDR55 may exert cell-type-specific defects. Such a possibility was suggested in zebrafish mutant *one-eyed pinhead (oep)*, where a ubiquitously expressed permissive EGF-related ligand Nodal co-receptor causes cell-type-specific abnormalities including cyclopia [51]. Biochemical analysis of WDR55 functions, especially in regard to the mechanisms modulating rRNA biosynthesis and the cell-type-specific susceptibility to the defect in WDR55, is awaited to prove this hypothesis.

Finally, this study shows that zebrafish *hi2786B* mutant that carries an insertional null mutation in *WDR55* locus exhibits developmental defects similar to *hkc* mutant in medaka, including defective formation of the thymus. The similarity of defects in medaka and zebrafish supports the possibility that the missense *hkc* mutation of WDR55 causes a severe deficiency of functional WDR55 similar to a null mutation, rather than retaining the partial functions of WDR55. On the other hand, a null mutation of WDR55 in mouse causes much earlier developmental arrest before implantation. The difference in developmental defects caused by WDR55 mutation between mouse and the two teleost species may be linked to the difference in the role of maternally inherited mRNA and proteins during embryogenesis. Maternal-to-zygotic transition of gene expression occurs as early as the 2-cell stage in mouse [52], whereas zygotic transcription in oviparous vertebrates occurs during blastula stage [41]. Our data further show that the half-life of WDR55 proteins synthesized in the cells is 7 to 10 days. Thus, it is possible that maternally inherited mRNA and proteins may contribute to the embryogenesis for substantially longer periods in oviparous vertebrate species including teleosts than in mammals. This difference between mouse and teleost should be carefully considered when genetic analysis using teleost species is implicated to understand mammalian development and human biology with medical goals.

In conclusion, we found that WDR55 is a novel modulator of nucleolar production of rRNA and the deficiency in WDR55 causes cell cycle arrest and developmental defects, including teleost thymus organogenesis. Further analysis of WDR55 function should lead to a better understanding of rRNA biosynthesis and vertebrate development.

## Materials and methods

### Animals

Medaka cab strain and cab-derived *hkc* mutant line were previously described [15]. Medaka kaga strain, which has a highly discrete genome sequence compared to cab strain, was used to map *hkc* mutation. p53-deficient mutant medaka line *p53^Y186X^* [38] and *rag1-EGFP* transgenic medaka line [30] were also used. Developmental stage was designated as described [53]. Where indicated, zebrafish *hi2786B* mutant strain [43] and *WDR55*-deficient mouse strain [44] were used. Animal experiments were performed with consent from the Animal Experimentation Committee of the University of Tokushima.

### Mapping of *hkc* Mutation

To identify the linkage group of *hkc* mutation, bulked segregant analysis was carried out on DNA isolated from 48 *hkc* embryos and 48 wild-type siblings derived from an *hkc*/cab×kaga mapping cross. Genetic mapping and chromosome walking on linkage group 18 were performed essentially as described by Geisler (2002) [54], using restriction fragment length polymorphism markers between cab and kaga strains. 407 *hkc* embryos were analyzed using markers described in MLBase (http://mbase.bioweb.ne.jp/dclust/ml_base.html) and additional markers that we identified. Scaffolds of medaka shotgun sequences were searched at Medaka Genome Project server (http://dolphin.lab.nig.ac.jp/medaka/). BAC library of medaka genomic DNA was previously described (Matsuda et al., 2001) [20]. Gene structure was predicted using Genscan (http://genes.mit.edu/GENSCAN.html).

### Morpholino Treatment of Medaka

50 µM of morpholino antisense oligonucleotides in 0.3x Danieu's solution containing 0.1% rhodamine-dextran was injected into fertilized cab eggs at 1-cell stage. The sequence of the morpholino was as follows: WDR55-splicing-inhibiting morpholino, 5′-AGA CTC CGT GTT CCT GAC CTT CAG-3′; and WDR55-translation-inhibiting morpholino, 5′-CCG CCA TGT TTG TTT GGT GAT TTT C-3′. Total RNA was extracted from the embryos at stage 25, and RT-PCR was carried out to confirm inhibition of mRNA splicing. Primers used for this RT-PCR were 5′-GGG CTA AAG CTG TTT AGC GT-3′ and 5′-GCC TCT CCC TTC CTC ATG TC-3′. Morphants were fixed at 5 or 6 dpf for *in situ* hybridization. The same amount of control morpholino (five-nucleotide substitution from the sequence specific for an unrelated gene *TC53327*) [30] did not affect the phenotype.

### mRNA Rescue in Medaka

cDNA fragments containing the entire coding region of WDR55 derived from either wild-type or *hkc* embryos were amplified using primers 5′-GCA GCT GTT CAG CGC AGA AG-3′ and 5′-AAC ACA ACT TTC CTG TCC AA-3′, and cloned into pCRII vector (Invitrogen). EcoRI fragments containing entire insert sequences were subcloned to pCSII+ expression vector [55]. 3′ ends of inserts were cut with NotI, and cDNA was transcribed using mMESSAGEmMACHINE Kit (Applied Biosystems). 10 ng/µl of WDR55 mRNA was injected into 1-cell-stage fertilized eggs derived from mating of *hkc*/+ heterozygotes. 10 ng/µl of EGFP mRNA was co-injected as internal control to confirm successful injection and translation.

### Cell Transplantation in Medaka

Adult thymocytes (5×10^2^) of *rag1-EGFP* transgenic medaka were labeled with CellTracker Orange CMTMR (Molecular Probes) and injected into sinus venosus of dechorionated embryos at 5 dpf. Thymic regions of recipients were observed under a laser scanning microscope at 1 day after the transplantation.

### Whole-Mount *In Situ* Hybridization and Alcian Blue Staining

Probes for detecting medaka *rag1*, *ikaros*, *tcrb*, *dlx2*, *pax9*, *gata1*, and *foxn1* were described previously [15],[30]. Sense and antisense probes for *WDR55* were produced from *WDR55* cDNA as described above and were labeled with digoxigenin using DIG RNA Labeling Kit (Roche). Plasmid containing zebrafish *rag1* [56] was a kind gift from Dr. C. E. Willett. Whole-mount *in situ* hybridization was carried out as described [15]. Alcian blue staining of cartilage structures was carried out as described [57].

### BrdU Labeling and Histological Analysis

Medaka embryos were soaked in 1 mg/ml BrdU in 0.03% sea salt water for 1.5 hours. Frozen sections (10 µm) were stained with anti-BrdU antibody and hematoxylin using BrdU In-Situ Detection Kit (BD Biosciences Pharmingen). Zebrafish larvae at 6 dpf were embedded in OCT compound (Sakura Finetek) and frozen. Five-micrometer sections were stained with hematoxylin and eosin. Cryosectioning and immunohistochemical staining of mouse fetal thymus were previously described [58].

### siRNA-Mediated Knockdown in Cells

siRNA specific for mouse WDR55 or a control siRNA (Invitrogen) was transfected into NIH3T3 cells using the protocol supplied by Invitrogen. At 44 hours after transfection, cells were either harvested for biochemical analysis or pulsed with 10 µM BrdU for 30 minutes, followed by staining with anti-BrdU antibody and 7-AAD (BD Biosciences Pharmingen). Three kinds of WDR55-specific siRNAs with different sequences gave similar results of varying degrees, so that the results from only one siRNA that demonstrated the strongest effects are shown.

### Intracellular Localization of *WDR55</EMPH>*

cDNA fragments of *WDR55* containing the entire open reading frame derived from either wild-type or *hkc* mutants were cloned into pEGFP-C1 (BD Biosciences Pharmingen). These constructs, pEGFP-WDR55WT (wild-type) and pEGFP-WDR55MT (*hkc* mutant), expressed fusion proteins that attached EGFP to the N-terminus of WDR55. Plasmid containing t-HcRed1-fibrillarin [59] was kindly provided by Dr. K. A. Lukyanov. pEGFP-WDR55WT, pEGFP-WDR55MT, or pEGFP-C1 was co-transfected with t-HcRed1-fibrillarin into 293T cells. At 30 hrs after transfection, fluorescence signals were detected with TCS SP2 laser scanning microscope (Leica).

In order to obtain the antibody specific for mouse WDR55, rabbits were immunized with a protein conjugated with a synthetic peptide of mouse WDR55 (Ac-CSSGHDQRLKFWDMTQLR-amide). NIH3T3 cells fixed with 4% PFA and permeabilized with 0.1% Triton-X were incubated with 1/300 dilution of anti-WDR55 antibody and 1/300 dilution of mouse anti-fibrillarin antibody (Abcam) for 1 hr. FITC-labeled goat anti-rabbit IgG antibody (Molecular Probes) and AlexaFluor633-labeled goat anti-mouse IgG antibody (Molecular Probes) were used for fluorescence visualization of antibody binding with a laser scanning microscope.

### RT-PCR

Total RNAs were extracted with Isogen (Wako Chemical) and cDNAs were synthesized using SuperscriptIII first strand synthesis system (Invitrogen). Quantitative RT-PCR was performed with SYBR premix ExTaq (Takara) and iCycler iQ Real Time PCR System (Bio-Rad). Amplified signals were confirmed to be single bands over gel electrophoresis, and normalized to the signals of medaka cytoplasmic actin, zebrafish β-actin, or mouse GAPDH. The primers used were as follows: medaka *WDR55*, 5′-GAC AGA TCC TCC AGA AAC GAA C-3′ and 5′-CAG GGT CCC TCT GTC ATC TC-3′; medaka *lck*, 5′-CGA ACA CTG CAA CTG TCC AA-3′ and 5′-ACA AGC TCC TTC AGC GAG TT-3′; medaka *p63*, 5′-CCA CGC TCA GAA CAA CGT GA-3′ and 5′-GAT CTG AAT GGG GCA CGT CT-3′; medaka *p73*, 5′-CAA TCC CCT CCA ACA CCG ATT-3′ and 5′-TCG TGA TTG GGG CAT CGT TTG-3′; zebrafish *WDR55*, 5′-AAA GAG CTC TGG TCA TCA GG-3′ and 5′-TAT CCC AAA CCT TCA GCG TT-3′; mouse *WDR55*, 5′-TCC ATC CGA CTC GAG ATC TG-3′ and 5′-GCC ATG TCG GCA ATG TAC TC-3′; and mouse *GAPDH*, 5′-CCG GTG CTG AGT ATG TCG TG-3′ and 5′-CAG TCT TCT GGG TGG CAG TG-3′. Other primers for medaka genes were described previously [15],[30],[37],[60]. Zebrafish *β-actin* primers and mouse *p21* primers were as described by Mathavan et al. (2005) [61] and Boley et al. (2002) [62], respectively. For the detection of maternal and embryonic *WDR55* expression, RT-PCR products were incubated with BstNI. BstNI cuts cab-derived but not kaga-derived WDR55 sequence. Primers used were 5′-GAC AGA TCC TCC AGA AAC GAA C-3′ and 5′-GCC GTC TCT TGA TGT TGA AGA C-3′.

### Northern Blot Hybridization

Total RNA was separated by electrophoresis in either 1% agarose gel containing MOPS or 6% polyacrylamide gel containing 8 M urea, and blotted onto positively charged nylon membrane (Biodyne Plus, Pall). After UV crosslinking, blotted total RNA was stained with methylene blue. The sequences of locked nucleic acid (LNA) probes conjugated with digoxigenin (DIG) at 3′ terminus are as follows: medaka and mouse 5.8S (probe used in Figure 3C and probe C in Figures 4A and E), 5′-tTC tTC aTC gAC gCA cGA gC-3′; medaka 18S, 5′-tAC tCC cCC cGG aAC cCA aA -3′ (probe A in Figure 4D); medaka ITS1, 5′-GtG CtG CtT CgC CaC GtT Cg-3′ (probe B in Figure 4B); and medaka ITS2, 5′-GaG CgG GgA AcA CcG AtT Ga-3′ (probe D in Figure 4D) (Greiner Bio-One). Small letters indicate LNAs. Medaka genomic DNA fragment containing 28S sequence (probe E in Figure 4D) was amplified with 5′-GAT TCC CAC TGT CCC TAC CT-3′ and 5′-AGA TCA AGC GAG CTT TTG CC-3′ primers. This DNA fragment was cloned in pCRII vector (Invitrogen), cut with XhoI, and transcribed using SP6 polymerase for DIG-labeled antisense probe (Roche DIG RNA Labeling Kit). Hybridization was carried out at 68°C and signals were detected with Gene Images CDP-Star Detection Kit (Amersham BioSciences).

### Antibody Blotting and Immunoprecipitation

Lysates of siRNA-transfected NIH3T3 in lysis buffer (50 mM Tris-HCl pH 8.0, 137 mM NaCl, 1% NP-40, 0.5% deoxycholate, and 0.1% SDS) with protease inhibitors were electrophoresed over SDS-PAGE and transferred to Immobilon-P membranes (Millipore). Membranes were incubated with 1/300 dilution of anti-WDR55 antibody or 1/300 dilution of rabbit anti-calnexin antibody (Santa Cruz), followed by horseradish-peroxidase-conjugated goat anti-rabbit IgG antibody (Santa Cruz), and visualized using ECL Plus Western Blotting Detection System (Amersham). Immunoprecipitation of U2OS cell lysates with anti-Pes1, anti-Bop1, and anti-WDR12 antibodies (Ascenion) was carried out as described [35].

### Accession Numbers

Database accession numbers from DDBJ/GenBank/EMBL for the genes identified in this study were as follows: medaka WDR55, AB372859; medaka lck, AB372860; medaka p63, AB372861; and medaka p73, AB372862.
