# Correction: WDR55 Is a Nucleolar Modulator of Ribosomal RNA Synthesis, Cell Cycle Progression, and Teleost Organ Development

**DOI:** 10.1371/annotation/6683988c-4647-4550-88e3-e8ba052f7916

**Published:** 2008-09-04

**Authors:** Norimasa Iwanami, Tomokazu Higuchi, Yumi Sasano, Toshinobu Fujiwara, Vu Q. Hoa, Minoru Okada, Sadiqur R. Talukder, Sanae Kunimatsu, Jie Li, Fumi Saito, Chitralekha Bhattacharya, Angabin Matin, Takashi Sasaki, Nobuyoshi Shimizu, Hiroshi Mitani, Heinz Himmelbauer, Akihiro Momoi, Hisato Kondoh, Makoto Furutani-Seiki, Yousuke Takahama

The following information was omitted from the Funding section: YT was supported by the Mitsubishi Foundation.

